# Plant community analysis along environmental gradients in moist afromontane forest of Gerba Dima, South-western Ethiopia

**DOI:** 10.1186/s12862-022-01964-4

**Published:** 2022-02-07

**Authors:** Abyot Dibaba, Teshome Soromessa, Bikila Warkineh

**Affiliations:** 1 College of Natural Sciences, Department of Biology, Debre Berhan University, P. O. Box 445, Debre Berhan, Ethiopia; 2 Center for Environmental Science, Addis Ababa University, P. O. Box 1176, Addis Ababa, Ethiopia; 3 College of Natural Sciences, Department of Plant Biology and Biodiversity Management, Addis Ababa University, P. O. Box 3434, Addis Ababa, Ethiopia

**Keywords:** Gerba Dima, Indicator species, Moist afromontane forest, Species diversity

## Abstract

**Background:**

This study was carried out in Gerba Dima Forest, South-Western Ethiopia, to determine the floristic composition, species diversity and community types along environmental gradients. Identifying and interpreting the structure of species assemblages is the main goal of plant community ecology. Investigation of forest community composition and structure is very useful in understanding the status of tree population, regeneration, and diversity for conservation purposes.

**Method:**

Ninety sample plots having a size of 25 × 25 m (625 m^2^) were laid by employing stratified random sampling. Nested plots were used to sample plants of different sizes and different environmental variables. All woody plant species with Diameter at breast height (DBH) ≥ 2.5 cm and height ≥ 1.5 m were recorded in 25 m × 25 m plots. Hierarchical (agglomerative) cluster analysis was performed using the free statistical software R version 3.6.1 using package cluster to classify the vegetation into plant community types. Redundancy Analysis (RDA) ordination was used in describing the pattern of plant communities along an environmental gradient.

**Result:**

One hundred and eighty plant species belonging to 145 genera, 69 families and comprising of 15 endemic species were recorded. Of these, 52 species (28.9%) were trees, 6 species (3.33%) were Trees/shrubs, 31 species (17.22%) were shrubs, 76 species (42.22%) were herbs, and 15 species (8.33%) were Lianas. *Rubiaceae*, *Acanthaceae* and *Asteraceae* were the richest family each represented by 11 genera and 11 species (6.11%), 9 genera and 11 species (6.11%), 6 genera and 11 species (6.11%), respectively of total floristic composition. Cluster analysis resulted in five different plant communities and this result was supported by the ordination result. RDA result showed altitude was the main environmental variable in determining the plant communities. The ANOVA test indicated that the five community types differ significantly from each other with regard to Electrical Conductivity and Potassium.

**Conclusions:**

Description of floristic diversity of species in Gerba Dima forest revealed the presence of high species diversity and richness. The presence of endemic plant species in the study forest shows the potential of the area for biodiversity conservation.

**Supplementary Information:**

The online version contains supplementary material available at 10.1186/s12862-022-01964-4.

## Background

Identifying and interpreting the structure of species assemblages is the main goal of plant community ecology. Gradients in species composition vis-à-vis either presence/absence or abundance data are commonly employed to evaluate community structure [1]. Legendre [2] distinguished between ‘true gradients’ in species composition, which are induced by environmental gradients, and ‘false gradients’, which may arise even in the absence of environmental heterogeneity as a result of biotic interactions within the community. Both true and false gradients may form distinct spatial patterns when mapped into geographic space. According to Seabloom et al. [3], different ecological processes create distinct spatial patterns, so that specific processes could be identified from their spatial signature. Hence, spatial analysis of community structure is of direct scientific interest, because spatial structures may be critical for identifying and understanding the underlying ecological processes [4]. Biotic filters determining limiting similarity is the assumed cause for species dissimilarity in traits within communities. Symmetric competitive interactions might indeed lead to the co-existence of ecologically distinct species, which minimize competition for shared resources (“symmetric competition” leading to limiting similarity [5].

The quest to explain the underlying processes for the assembly of local communities is still a major focus in plant community ecology, as researchers keep examining them through both observational and experimental studies [6]. The multidimensional ecological niche space determines the distribution of a species within a community [7]. Physiographic and edaphic factors can determine which plant species will colonize a site since plant species vary in their tolerance and utilization of resources site [8]. These variations have been regarded as a driving force for the coexistence of species in a similar environment [9] and can explain broad-scale compositional differences among multiple resource gradients [10, 11]. The upper storey tree density as an abiotic factor can also affect community composition as understorey species differ in their ability to tolerate stresses imposed by competitive trees [12, 13]. Moreover, by increasing the abundance of annual and biennial plants, disturbances can affect community composition via favouring stress-tolerant species [13, 14].

Information on species composition and diversity of tree species plays a pivotal role not only to understand the structure of a forest community but also in planning and implementation of conservation strategy of the community [15]. Investigation of forest community composition and structure is very useful in understanding the status of tree population, regeneration, and diversity for conservation purposes [16]. Quantitative information on composition, distribution, and abundance of woody species has paramount importance in understanding the form and structure of a forest community and for planning and implementation of conservation strategy of the community.

The recent data on forest resources of Ethiopia reported in FAO [17] puts Ethiopia among countries with a forest cover of 10–30%. According to this report, Ethiopia’s forest cover (FAO definition) is 12.2 million ha (11%). It further indicated that the forest cover shows a decline from 15.11 million ha in 1990 to 12.2 million ha in 2010, during which 2.65% of the forest cover was deforested. This study was conducted in the Gerba Dima forest found in South-Western Ethiopia with the aim of investigating the species composition, species diversity, community types and to relate the distribution of plant community types to some environmental parameters.

## Methods

### The study area

**Fig. 1:**
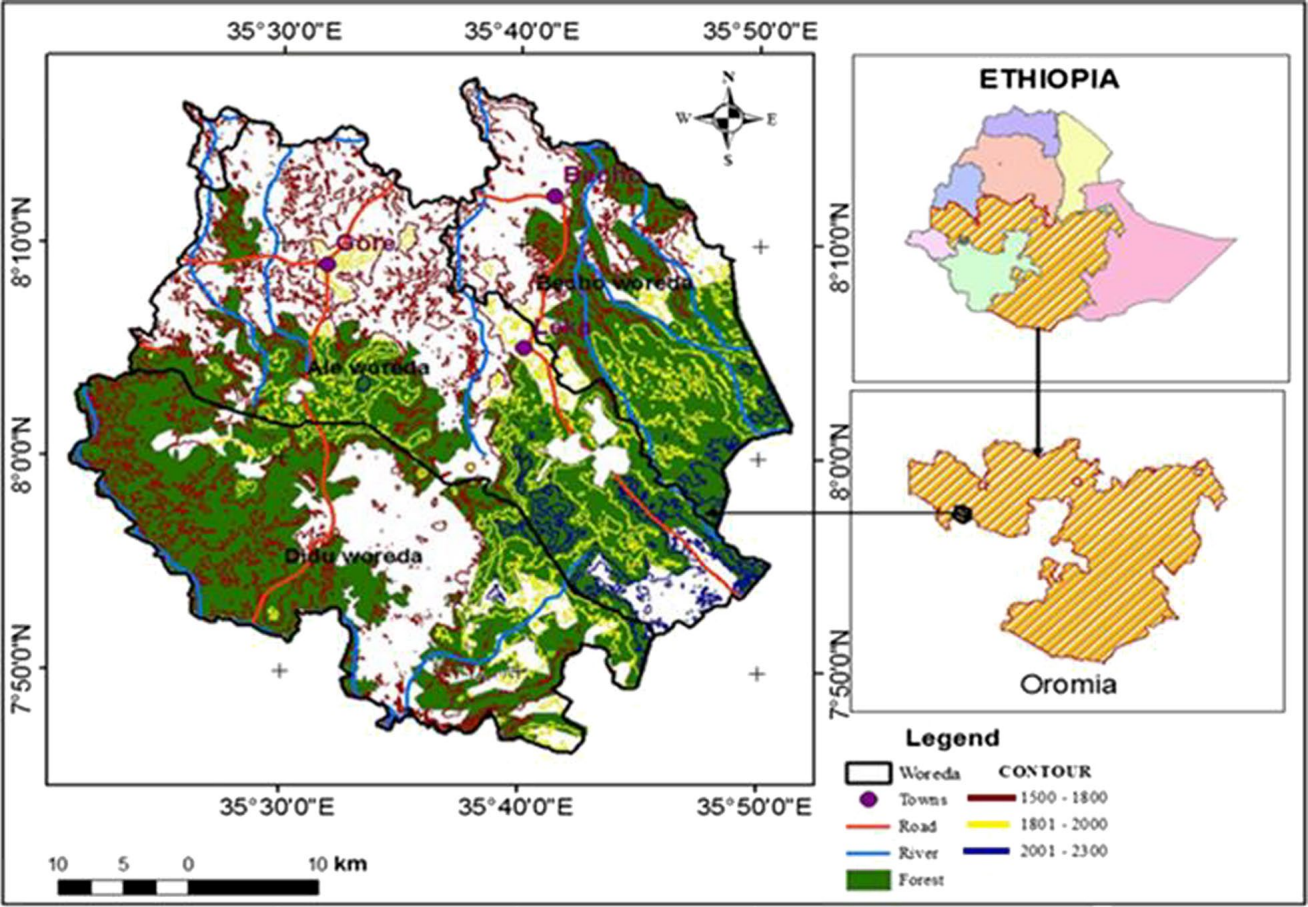
Map of Ethiopia, Oromia region and Gerba Dima forest

This study was carried out in the Gerba Dima forest found in the Illu Aba Bora zone of Oromia regional state of Ethiopia and located between 7° 45' to 8° 10' North latitude and 35° 29' to 35° 50' East longitude. The study forest is bounded by Baro River to the south and west direction whiles three other rivers, namely Bote, Hoyi and Sor cross part of the forest in the east (Fig. 1). The geology of the study site is characterized by the Underlying basement rock consisting of intensively folded and faulted Precambrian rocks, overlain by Mesozoic marine strata and Tertiary basalt types [18]. The main soil types of the study area are red or brownish ferrisols derived from the volcanic parent material. Other soil groups in the area include nitosols, acrisols, vertisols, and cambisols soil types exist in the study site [19].

The rainfall data collected from the nearest Gore meteorological station to the study forest indicated that the study area receives very high annual rainfall and characterized by unimodal rainfall pattern, which shows low rainfall in December, January and February, gradually increasing to the peak period in August. The mean annual rainfall of 1854 mm while the monthly mean maximum and mean minimum temperature of the area is 27.2 ℃ and 13.3 ℃, respectively. The mean annual temperature is 19.2 ℃ and with slight variation from year to year [20].

**Fig. 2:**
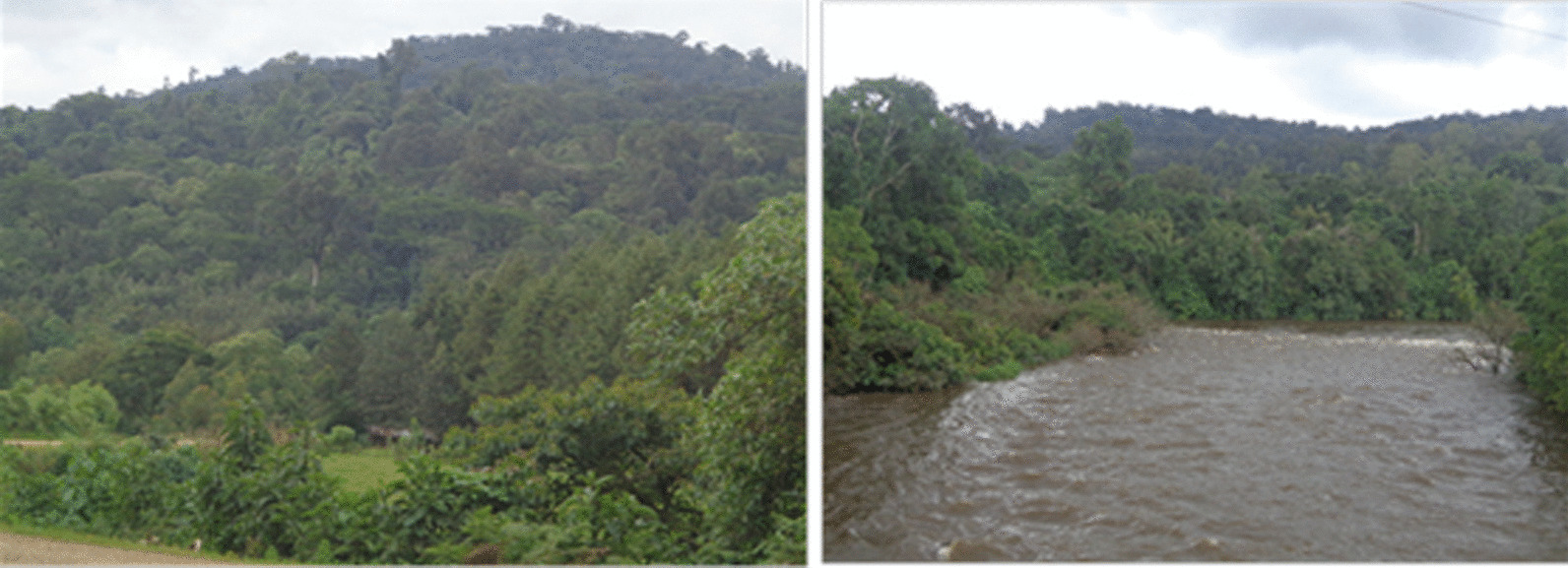
Photograph illustrating the forest overview

The vegetation type at Gerba Dima is part of the moist evergreen afromontane forest with characteristic emergent species that form the upper canopy includes *Pouteria adolfi-friederici* (Fig. 2). *Albizia gummifera, A. schimperiana, A. grandibracteata, Sapium ellipticum, Euphorbia ampliphylla, Ekebergia capensis, Ficus sur, Hallea rubrostipulata, Ocotea kenyensis, Olea welwitschii, Polyscias fulva and Schefflera abyssinica* are other characteristic species of this vegetation type [21].

### Sampling method

**Fig. 3:**
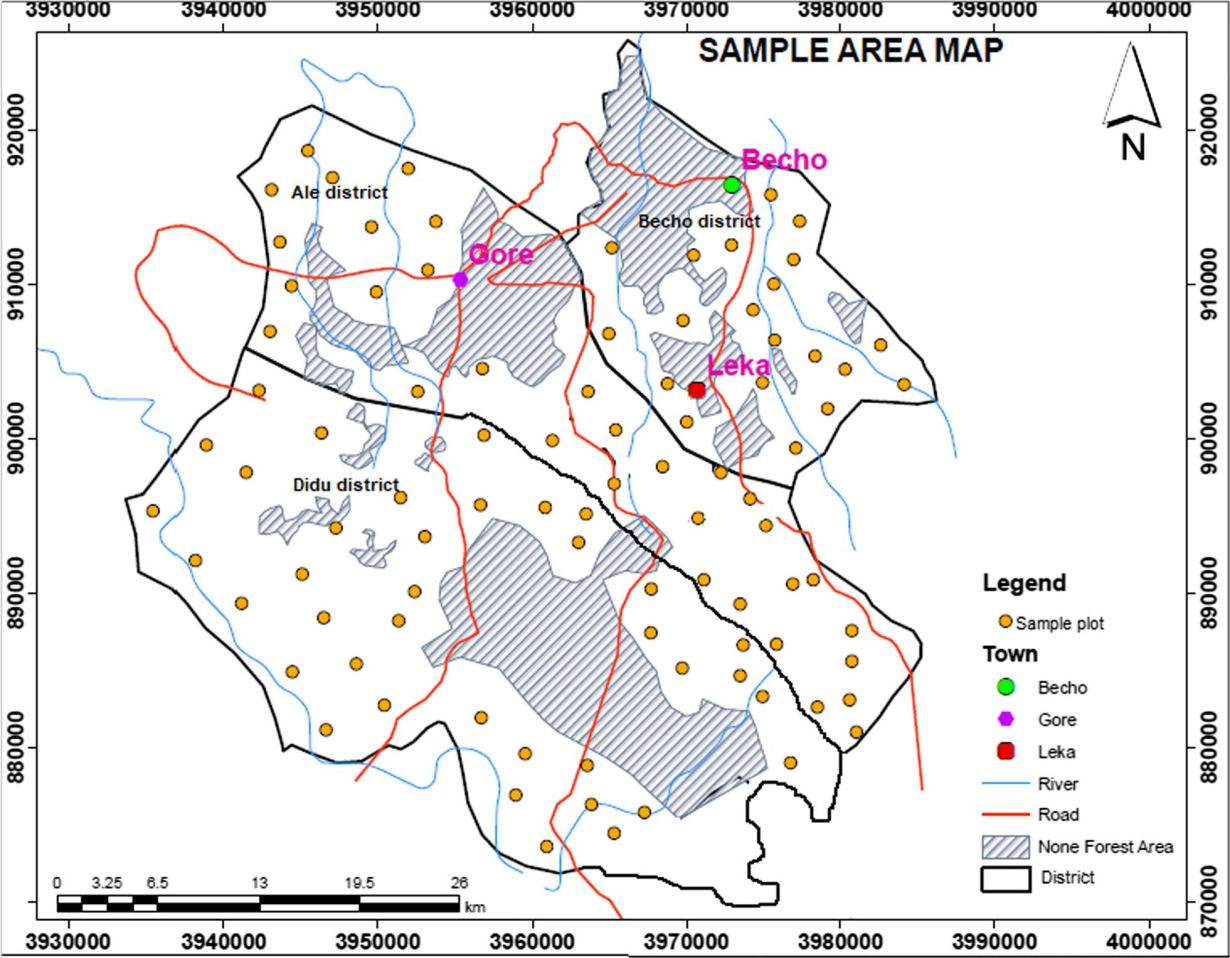
A Map showing the distribution of sample plots

In this study, a stratified random sampling design was used to collect vegetation and environmental data [1, 22]. Using Arc GIS version 10.3, the study forest was stratified based on the altitudinal gradient and three types of strata in the form of contour were established. Strata one was distributed between 1500 and 1800 m altitudinal ranges whereas strata two and three were found between 1801–2000 m and 2001–2300 m altitudinal ranges respectively (Fig. 1). Sample plots were assigned in each contour in the form of Random points Using Arc GIS version 10.3 (Fig. 3).

Ninety sample plots having a size of 25 × 25 m (625 m^2^) along each contour were laid. Nested plots were used to sample plants of different sizes and different environmental variables. All woody plant species with Diameter at breast height (DBH) ≥ 2.5 cm and height ≥ 1.5 m were recorded in 25 m × 25 m plots. Within the major plots, five 3 m × 3 m subplots (9m^2^) was used to collect shrubs with dbh < 2.5 cm and > 1.5 m height. Within each 9 m^2^ subplots, two 1 m^2^ subplots were used to collect data on the species and abundance of herbaceous plants. Finally, the percent cover of all plant species found within the sample plot was visually estimated and converted to the Braun-Blanquet scale as modified by [23]. Every plant species encountered in each plot were recorded. Plant specimens were collected, pressed, dried and brought to the National Herbarium (ETH), Addis Ababa University for taxonomic identification. The specimens were determined by comparing with authenticated specimens housed at ETH and by referring to published volumes of Flora of Ethiopia and Eritrea [24–32].

Physiographical variables, namely altitude, geographic coordinates, slope and aspect, were recorded for each quadrat using GPS, Clinometer and Compass respectively. The values for aspect were codified based on Woldu [33], where N = 0, NE = 1, E = 2, SE = 3, S = 4, SW = 3.25, W = 2.5, NW = 1.25 before analysis. For each sample plot, a disturbance was determined on the basis of a five point scale following [34]. The five scales of disturbance scores were based on visible signs of tree cutting, grazing and presence of beehives. The points of scale were 0 = (No disturbance), 1 = (0–20% of the quadrat disturbed), 2 = (21–40% of the quadrat disturbed), 3 = (41–60% of the quadrat disturbed), 4 = (61- 80% of the quadrat disturbed), 5 = (81–100% of the quadrat disturbed).

For analysing soil variables, soil samples were collected with a soil core sampler from the top 40 cm depth within 1 m × 1 m subplots at the four corners and middle of the quadrat. Composite soil samples from samples collected from the four corners and the middle of quadrats were brought to the soil laboratories of Addis Ababa University (AAU). The soil samples were air-dried, rolled and passed through a 2 mm sieve for laboratory analyses. These soil samples were analysed for pH, electrical conductivity (EC), sodium, potassium, organic matter, total nitrogen, available phosphorus and texture following standard procedures outlined in [35]. The pH and EC were measured using a pH meter and EC meter in the supernatant suspension of 1:2.5 soil–distilled water mixtures. Available Sodium and Potassium were determined using a flame photometer. Organic matter was determined by the ignition method. The texture was determined on the basis of Bouycous Hydrometer method with the categories sand, silt, and clay (expressed as % weight) while total nitrogen was determined using Kjeldhal method. Available Phosphorus was determined by the Bray-I method and the absorbance of the Bray-I extract is measured at 882 nm in a spectrophotometer.

### Data analysis

In this study, hierarchical (agglomerative) cluster analysis was performed using the free statistical software R version 3.6.1 [36] using package cluster to classify the vegetation into plant community types. The similarity ratio with Ward's group linkage method was applied for cluster analysis i.e. to determine plots that can be classified into the same groups based on the species abundance data. The decision on the number of groups (clusters) was based on objective methods of obtaining an optimal number of clusters, the Multi Response Permutation Procedures (MRPP) technique (no-difference hypothesis) and the ecological interpretation of the groups conducted in R program. The T and A statistic of MRPP output were used to obtain the number of clusters. The test statistic T describes the separation between the groups. The more negative T value, the stronger the separation. The P-value associated with T is determined by numerical integration of the Pearson type III distribution. The P-value is useful for evaluating how likely an observed difference is due to chance [37]. The agreement statistic A describes within-group homogeneity, compared to the random expectation, and falls between 0 and 1. When all items within-groups are identical A = 1 and 0 if the groups are heterogeneous. In community ecology, A values are commonly below 0.1, and an A value greater 0.3 is fairly high [37].

From the output of the objective method, a sharp bend at the specific cluster in the plot could be a good indication of the number of clusters in the data [38]. The community types identified from the cluster analysis were further refined in a synoptic table where species occurrences were summarized as synoptic cover-abundance values [39]. Dominant species of each community type were identified based on their synoptic values and community types were named after one or more dominant species. The identified groups were tested for the hypothesis of no difference between the groups (clusters) using nonparametric Multi-Response Permutation Procedure (MRPP). Indicator species analysis was performed in R using package labdsv. Indicator values were tested for statistical significance using a randomization (Monte Carlo) technique. Species richness, evenness, Shannon diversity and evenness indices were computed using the free statistical software R version 3.6.1 [36]. The Shannon diversity index (H') was calculated from the equation:

$$H^{\prime} = - \sum\limits_{i = 1}^{s} {pi\,ln pi}$$

where pi, is the proportion of individuals found in the ith species. The values of the Shannon diversity index is usually found to fall between 1.5 and 3.5 and only rarely surpasses 4.5 [1, 39]. The Shannon evenness index (J) was calculated from the ratio of observed diversity to maximum diversity using the equation:

$$J = \frac{{H^{\prime}}}{H\,max} = \frac{{H^{\prime}}}{ln\,s}$$

where Hmax is the maximum level of diversity possible within a given population, which equals ln (number of species). J is normal between 0 and 1, and with 1 representing a situation in which all species are equally abundant [40].

Information about endemic species, their habit, IUCN status and geographical distributions was determined by referring to [25–33, 42].

In this study, Redundancy Analysis (RDA) ordination was used in describing the pattern of plant communities along an environmental gradient since the preliminary analysis of the vegetation data using Deterended Correspondence Analysis (DCA) revealed that the longest axis of DCA for the dataset was less than 3 (= 2.22). Before the application of RDA ordination, environmental variables, which were relatively more important in explaining the species data, were selected using the Monte Carlo technique and function Adonis test for their significance. Computation of variance inflation factor (vif) was also conducted to eliminate those environmental variables that are collinear. The community types obtained were subjected to an ANOVA based on environmental variables to find out whether there are significant variations between the groups. Pearson's product-moment correlation coefficient was calculated to evaluate the relationship between the environmental variables.

## Results

### Floristic composition

One hundred and eighty (180) plant species belonging to 145 genera and 69 families were recorded and identified in the sample plots in the Gerba Dima forest (Table 1). Of these, 52 species (28.9%) were trees, 6 species (3.33%) were Trees/shrubs, 31 species (17.22%) were shrubs, 76 species (42.22%) were herbs, and 15 species (8.33%) were Lianas. Angiosperms were represented by 160 species while the rest 20 species were Pteridophytes. Among Angiosperms, *Rubiaceae*, *Acanthaceae* and *Asteraceae* were the richest family each represented by 11 genera and 11 species (6.11%), 9 genera and 11 species (6.11%), 6 genera and 11 species (6.11%), respectively of total floristic composition, followed by *Fabaceae* 8 genera and 9 species (5%), *Euphorbiaceae* 6 genera and 7 species (3.89%). The remaining families represented less than 3% of species each. Eleven families, 13 genera and 20 species represented pteridophytes. *Aspleniaceae*, *Dryopteridaceae* and *Pteridaceae* were the richest Pteridophytes represented by 6, 3 and 2 species respectively. The genus *Vernonia, Ficus*, *Asparagus*, *Dracaena *were represented by 5,4,3,3 species respectively and *Aframomum*, *Albizia*, *Asparagus*, *Cyperus*, *Euphorbia*, *Hippocratea*, *Hypoestes*, *Justicia*, *Maytenus*, *Olea*, *Peperomia*, *Polyscias*, *Pteris*, *Rubus*, *Schefflera*, *Solanecio*, *Solanum, Tacazzea*, and *Zehneria* were represented by 2 species each and the rest genera contained a single species each.

**Table 1:** List of species in Gerba Dima Forest

No	Scientific names	Family	Local names^a^	Habit	Voucher No.
1	*Acanthopale ethio-germanica* Ensermu	Acanthaceae	Dargu	S	AD005
2	*Acanthus eminens* C.B.Clarke	Acanthaceae	Qosambe booyyee	S	AD107
3	*Achyranthes aspera* L	Amaranthaceae	Maxxane	H	AD160
4	*Achyrospermum schimperi* (Hochst. ex Briq.) Perkins	Lamiaceae	–	H	AD134
5	*Adiantum poiretii* Wikstr	Adiantaceae		H	AD120
6	*Aerangis brachycarpa* (A. Rich) Th Dur.& Schinz	Orchidaceae	–	H	AD062
7	*Aframomum corrorima* (Braun) Jansen	Zingiberaceae	Ogiiyo	H	AD045
8	*Aframomum zambesiacum* (Baker) K. Schum	Zingiberaceae	Ogiiyo jaldessaa	H	AD096
9	*Ageratum conyzoides* L	Asteraceae	–	H	AD038
10	*Ajuga sp*. (= Friis et al. 1456)	Lamiaceae	Gondii	H	AD118
11	*Alangium chinense* (Lour.) Harms	Alengeaceae	Hudu fardaa/sendo	T	AD007
12	*Albizia gummifera* (J.F. Gmel.) C.A. Sm.,	Fabaceae	Ambabbessa dhaltu	T	AD078
13	*Albizia schimperiana* Oliv	Fabaceae	Ambabbessa kormaa	T	AD009
14	*Alchemilla abyssinica* Fresen	Roseaceae	Korbesso	H	AD013
15	*Allophyllus abyssinicus* (Hochst.) Radlk	Sapindaceae	Se'o	T	AD021
16	*Antrophyum mannianum* Hook	Vittariaceae	Gixoo	H	AD082
17	*Apodytes dimidiata* E. Mey. ex Arn	Icaccinaceae	Wandabiyo	T	AD123
18	*Arisaema mooneyanum* Gilbert & Mayo	Araceae	Kiicu	H	AD144
19	*Asparagus africanus* Lam	Asparagaceae	Sariiti	H	AD165
20	*Asparagus flagellaris* (Kunth) Baker	Asparagaceae	Sariiti	H	AD180
21	*Asparagus setaceus* (Kunth) Jessop	Asparagaceae	Sariiti	H	AD174
22	*Asplenium aethiopicum* (Burm.f.) Bech	Aspleniaceae	–	H	AD179
24	*Asplenium bugoiense* Hieron	Aspleniaceae	Giixoo	H	AD143
23	*Asplenium elllottii* C.H.Wright,	Aspleniaceae	Giixoo	H	AD122
25	*Asplenium erectum* Bory ex Willd	Aspleniaceae	–	H	AD101
27	*Asplenium sandersonii* Hook	Aspleniaceae	Giixoo	H	AD083
26	*Asplenium warnetkei* Hieron	Aspleniaceae	Giixoo	H	AD042
28	*Bersama abyssinica* Fresen	Melianthaceae	Lolchisaa	T	AD024
29	*Bothriocline schimperi* Oliv. & Hiern exBenth	Asteraceae	Ilbu	S	AD129
30	*Brillantaisia madagascariensis* T. Anders. ex Lindau	Acanthaceae	Huxii	S	AD037
31	*Brucea antidysenterica* J. F. Mill	Simaroubaceae	Qomanyo	T	AD041
32	*Canthium oligocarpum* Hiern	Rubiaceae	Mixo	S	AD029
33	*Cassipourea malosana* (Baker) Alston	Rhizophoraceae	Looko	T	AD046
34	*Cayratia gracilis* (Guill. & Perr.) Suesseng	Vitaceae	Kalaalaa qamale	H	AD093
35	*Celtis africana* Burm.f	Ulmaceae	Ceeyii	T	AD015
36	*Chionanthus mildbraedii* (Gilg & Schellenb.) Stearn	Oleaceae	Kara waayyu	T	AD004
37	*Cissampelos mucronata* A.Rich	Menispermaceae	–	L	AD008
38	*Clausena anisata* (Wild.) Benth	Rutaceae	Ulmaayye	S	AD087
39	*Clerodendrum myricoides* (Hochst.) Varlee,	Lamiaceae	Maraasisaa	S	AD099
40	*Clematis longicauda* Steud. ex A. Rich	Ranuaculaceae	Fiitii	L	AD002
42	*Coffea arabica* L	Rubiaceae	Buna	T/S	AD010
41	*Coleochloa abyssinica* (Hochsl. ex A Rick) Gilly	Cyperaceae	Coqorsa mukaa	H	AD019
43	*Combretum paniculatum* Vent	Combreataceae	Bagge	L	AD177
44	*Commelina diffusa* Burm.f	Commelinaceae	Qorxabo	H	AD161
45	*Coniogramme africana* Heiron	Hemionitidaceae	–	H	AD006
46	*Cordia africana* Lam	Boraginaceae	Waddessa	T	AD110
47	*Crotalaria rosenii* (Pax) Milne-Redh. ex Polhill	Fabaceae	Ceekaa	H	AD147
48	*Croton macrostachyus* Del	Euphorbiaceae	Makkanisa	T	AD030
49	*Cucumis dipsaceus* Ehrenb. ex Spach	Cucurbitaceae	Umbaa'oo	H	AD036
50	*Culcasia falcifolia* Engl	Araceae	Qasso	H	AD077
51	*Cyathea manniana* Hook	Cyatheaceae	Sesino	T	AD074
52	*Cyperus fischerianus* A. Rich	Cyperaceae	Qunni	H	AD126
53	*Cyperus longus* L	Cyperaceae	–	H	AD011
54	*Dalbergia lactea* Vatke	Fabaceae	Sarxe dhittaa	S	AD018
55	*Deinbollia kilimandscharica* Taub	Sapindaceae	Qaso	T	AD017
56	*Desmodium repandum* (Vahl)DC	Fabaceae	Maxxanne	H	AD033
57	*Didymochlaena truncatula* (Sw.)J.Sm	Dryopteridaceae	–	H	AD035
58	*Dombeya torrida* (J.F. Gmel.) P.Bamps	Sterculiaceae	Daanisaa	S	AD034
63	*Doryopteris concolor* (Langsd & Fisch.) Kuhn in von der Deck.efl	Dryopteridaceae	–	H	AD051
59	*Dracaena afromontana* Mildbr	Dracenaceae	Sarxe	T/S	AD072
60	*Dracaena fragrans* (L.) Ker Gawl	Dracenaceae	Sarxe	S	AD090
61	*Dracaena steudneri* Engl	Dracenaceae	Sarxe	T	AD108
62	*Drynaria volkensii* Hieron	Polypodiaceae	Balessa	H	AD012
64	*Ehretia cymosa* Thonn	Boraginaceae	Ulaagaa	T	AD026
65	*Ekebergia capensis* Sparrm	Meliaceae	Sombo	T	AD061
66	*Elaeodendron buchananii* (Loes.) Loes	Celastraceae	Waaso	T	AD167
67	*Elastostema monticolum* Hook.f	Urticaceae	–	H	AD162
68	*Ensete ventericosum* (Welw.) Cheesman	Musaceae	Eeppoo	H	AD171
69	*Erythrococca trichogyne* (Muell. Arg.) Prain	Euphorbiaceae	Caakkoo	T/S	AD032
70	*Euphorbia ampliphylla* Pax	Euphorbiaceae	Adaami	T	AD040
71	*Euphorbia schimperiana* Scheele	Euphorbiaceae	Ananno	S	AD064
72	*Ficus exasperata* Vahl	Moraceae	Baalaantaayii	T	AD068
73	*Ficus ovata* Vahl	Moraceae	Qilxu	T	AD065
74	*Ficus sur* Forssk	Moraceae	Harbu	T	AD073
75	*Ficus thonningii* Blume	Moraceae	Dambii	T	AD136
76	*Flacourtia indica* (Burm.f.) Merr	Flacourtiaceae	Akuku	T	AD139
77	*Galiniera saxifraga* (Hochst.) Bridson	Rubiaceae	Simararu	T	AD137
78	*Glycine wightii (*Wight '& Am) Verde	Fabaceae	Kalaalaa	H	AD170
79	*Gouania longispicata* Engl	Rhaminaceae	Hidda reffaa	L	AD020
80	*Hallea rubrostipulata* (K. Schum.) J.-F. Leroy	Rubiaceae	Oobo/Bootto	T	AD016
81	*Hibiscus panduriformis* Burm.f	Malviaceae	Dabbasee	H	AD163
82	*Hippocratea africana* (Willd.) Loes	Celastraceae	Xiyo	L	AD166
83	*Hippocratea pallens* Planch ex Oliver	Celastraceae	Qawo	L	AD121
84	*Hypoestes forskaolii* (Vahl) R. Br	Acanthaceae	Dargu	H	AD124
85	*Hypoestes triflora* (Forssk.) Roem & Schult	Acanthaceae	Dargu	H	AD135
86	*Ilex mitis* (L.) Radlk	Aquifoliaceae	Qato	T	AD155
87	*Ipomea indica* (Burm. f) Merrill	Convolvulaceae	Kalaalaa	H	AD148
88	*Isoglossa somalensis* Lindau	Acanthaceae	Ilbu	H	AD001
89	*Jasminum abyssinicum* Hochst. ex DC	Oleaceae	Ilchime	L	AD080
90	*Justicia bizuneshiae* Ensermu	Acanthaceae	-	H	AD059
91	*Justicia schimperiana* (Hochst. ex Nees) T. Anders	Acanthaceae	Dhumugaa	S	AD053
92	*Kalanchoe petitiana* A. Rich	Crassulaceae	Bosoqe mukaa	H	AD044
93	*Keetia gueinzii* (Sond.) Bridson	Rubiaceae	Halale	T/S	AD111
94	*Lagera crispata* (Vahl) Hepper & Wood	Asteracea	–	H	AD117
95	*Landolphia buchananii* (Hall.f.) Stapf	Apocynaceae	Geebbo	L	AD133
96	*Lepidotrichilia volkensii* (Gurke) Leory	Meliaceae	Haalalee	T	AD138
97	*Lobelia giberroa* Hemsl	Lobeliaceae	Dingiraro	S	AD169
98	*Loxogramme abyssinica* (Baker) MG. Price	Polypodiaceae	Giixo	H	AD175
99	*Macaranga capensis* (Baill.) Sim	Euphorbiaceae	Ongo	T	AD168
100	*Maesa lanceolata* Forssk	Myrsinaceae	Abbayyi	T	AD027
101	*Marattia fraxinea* Sm	Marattiaceae	–	H	AD028
102	*Maytenus gracilipes* (Welw.ex Oliv.) Exell	Celastraceae	Kombolcha	S	AD114
103	*Maytenus undata* (Thunb.) Blakelock	Celastraceae	Ilikke	T	AD132
104	*Megalastrum lanuginosum* (Willd. ex Kaulf) Holttum	Tectariaceae	–	H	AD151
105	*Microglossa pyriflolia* (Lam.) O. Kuntze	Asteraceae	Nobbe	H	AD173
106	*Millettia ferruginea* (Hochst.) Baker	Fabaceae	Sottallo	T	AD131
107	*Monothecium glandulosum* Hochst	Acanthaceae	Dargu	H	AD091
108	*Myrsine africana* L	Myrsinaceae	–	S	AD089
109	*Ocimum lamiifolium* Hochst.ex Benth	Lamiaceae	Damakase	S	AD097
110	*Olea capensis* L	Oleaceae	Gagamaa	T	AD100
111	*Olea welwitschii* (Knobl.) Gilg & Schellenb	Oleaceae	Ba'aa	T	AD050
112	*Oplismenus hirtellus* (L.) P. Beauv	Poaceae	Sutto gogorrii	H	AD092
113	*Oxyanthus speciosus* DC	Rubiaceae	Abraango jaldessaa	T/S	AD079
114	*Pavonia schimperiana* Hochst. ex A. Rich	Malvaceae	Gajjo	H	AD084
115	*Pentas schimperiana* (A. Rich.) Vatke	Rubiaceae	–	H	AD031
116	*Peperomia abyssinica* Miq	Piperaceae	Sarxe mukaa	H	AD176
117	*Peperomia retusa* (L.f.) A. Dietr	Piperaceae	–	H	AD130
118	*Peponium vogelii* (Hook.f.) Engl	Cucurbitaceae	Tojjo	H	AD066
119	*Phaulopsis imbricata* (Forssk.) Sweet	Acanthaceae	Dargu	H	AD039
120	*Phoenix reclinata* Jacq	Araceae	Mexxi	T	AD022
121	*Phyllanthus sepialis* Muell. Arg	Euphorbiaceae	Qacamaa	S	AD172
122	*Pilea rivularis* Wedd	Urticaceae	–	H	AD153
123	*Piper capense* L.f	Piperaceae	Tunjo	H	AD014
124	*Pittosporum viridiflorum* Sims	Pittosporaceae	Soolee	T	AD070
125	*Polyscias farinosa* (Del.) Harms	Araliaceae	–	T	AD095
126	*Polyscias fulva* (Hiern) Harms	Araliaceae	Karaso	T	AD119
127	*Polystachya rivae* Shweinf	Orchidaceae	Capho	H	AD094
128	*Polystichum wilsonii* H. Christ	Dryopteridaceae	–	H	AD113
129	*Pouteria adolfi-friederici* (Engl.) Baehni	Sapotaceae	Qararo	T	AD152
130	*Premna schimperi* Engl	Verbenaceae	Urgessaa	S	AD178
131	*Prunus africana* (Hook. f.) Kalkm	Roseaceae	Homii	T	AD159
132	*Psychotria orophila* Petit	Rubiaceae	Xumaane	S	AD025
133	*Pteris dentata* Forssk	Pteridaceae	Giixoo	H	AD157
134	*Pteris pteridioides* (Hook.) ballard	Pteridaceae	Giixoo	H	AD076
135	*Pterolobium stellatum* (Forssk.) Brenan	Fabaceae	Harangamaa	S	AD154
136	*Pupalia micrantha* Haumam	Amaranthaceae	Maxxanne	H	AD128
137	*Ranunculus multifidus* Forssk	Ranunculaceae	–	H	AD149
138	*Rhamnus prinoides* L'Herit	Rhamnaceae	Gesho	S	AD067
139	*Ritchiea albersii* Gilg	Capparidaceae	Daqqo	T	AD140
140	*Rothmannia urcelliformis* (Hiern) Robyns	Rubiaceae	Diibo	T	AD069
141	*Rubus apetalus* Poir	Roseaceae	Goraa	S	AD075
142	*Rubus steudneri* Schweinf	Roseaceae	Goraa	S	AD071
143	*Rytigynia neglecta* (Hirn) Robyns	Rubiaceae	Mixo	S	AD112
144	*Sapium ellipticum* (Krauss) Pax	Euphorbiaceae	Bosoqa	T	AD109
145	*Scadoxus nutans* (Friis & J. Bjørnstad) Friis & Nordal	Amaryllidaceae	Qulubi jaldessaa	H	AD088
146	*Schefflera abyssinica* (Hochst. ex A. Rich.) Harms	Araliaceae	Gatamaa	T	AD104
147	*Schefflera myriantha* (Bak.) Drake	Araliaceae	Qero	L	AD086
148	*Sericostachys scandens* Gilg & Lopr	Amaranthaceae	Suddi	L	AD106
149	*Setaria megaphylla* (Steud.) Th. Dur. & Schinz	Poaceae	Gowaa	H	AD058
150	*Solanaceo manni* (Hook.f.) C. Jeffrey	Asteraceae	Rejjii caakkaa	S	AD125
151	*Solanecio gigas* (Vatke) C. Jeffrey	Asteraceae	Raafu boyye	S	AD054
152	*Solanum adoense* Hochst. ex A. Rich	Solanaceae	Hiddi- xino	S	AD102
153	*Solanum giganteum* Jacq	Solanaceae	Tambo arbaa	S	AD048
154	*Stellaria mannii* Hook.f	Caryophyllaceae	Moccoo	H	AD127
155	*Syzygium guineense* (Willd.) DC	Myrtaceae	Baddessaa	T	AD141
156	*Tacazzea apiculata* Oliv	Asclepidiaceae	Gebbo	L	AD116
157	*Tacazzea conferta* N.E. Br	Asclepidiaceae	Gebbo qalame	L	AD145
158	*Teclea nobilis* Del	Rutaceae	Mola'ee	T	AD158
159	*Tectaria gemmifera* (Fee) Alston	Tectariaceae	Gixoo	H	AD023
160	*Thalictrum rhynchocarpum* Dill. & A. Rich	Ranunculaceae	Finge	H	AD105
161	*Thunbergia alata* Boj. ex Sims	Acanthaceae	–	H	AD043
162	*Tiliacora troupinii* Cufod	Menispermaceae	Liqixi	L	AD146
163	*Trema orientalis* (L.) Bl	Ulmaceae	Huddu farddaa	T	AD164
164	*Trichilia dregeana* Sond	Meliaceae	Luyyaa	T	AD049
165	*Trifolium rueppellianum* Fresen	Fabaceae	Amagixa	H	AD150
166	*Trilepisium madagascariense* DC	Moraceae	Same'eko/ceeyii	T	AD085
167	*Tristemma mauritianum* J. F. Gmel	Melistostomaceae	–	H	AD052
168	*Triumfetta brachyceras* K. Schum	Tilaceae	Incciinii	S	AD142
169	*Turraea holstii* Gurke	Meliaceae	Ceekaa	S	AD003
170	*Urera hypselodendron* (A. Rich.) Wedd	Urticaceae	Capho	L	AD047
171	*Urtica simensis* Steudel	Urticaceae	Doobbii	H	AD115
172	*Vangueria apiculata* K. Schum	Rubiaceae	–	T	AD056
173	*Vepris dainellii* (Pichi-Serm.) Kokwaro	Rutaceae	Hadhessa	T	AD098
174	*Vernonia amygdalina* Del	Asteraceae	Eebicha	T	AD055
175	*Vernonia auriculifera* Hiern	Asteraceae	Rejjii	T/S	AD156
176	*Vernonia hochstetteri* Sch. Bip. ex Walp	Asteraceae	Soyama masango	S	AD057
177	*Vernonia rueppellii* Sch. Bip. ex Walp	Asteraceae	Tambo Arbaa	S	AD103
178	*Vernonia wollastonii* S. Moore	Asteraceae	–	H	AD060
179	*Zehneria minutiflora* (Cogn) C. Jeffrey	Cucurbitaceae	Kalaalaa bosonu	H	AD063
180	*Zehneria scabra* (Linn. f) Sond	Cucurbitaceae	Kalaalaa bosonu	H	AD081

^a^Local name = Afan Oromo

Based on the information available on the published Floras of Ethiopia a total of 15 endemic plant species in 11 families were recorded (Table 2), comprising more than 8.33% of the recorded species. *Asteraceae* was the first family having three endemic species, followed by *Acanthaceae* and *Fabaceae* (two species each). The remaining eight families have a single species each in the endemic species list. Among the total endemic species, herb, tree, shrub and liana growth forms were represented by 6,3,4,2 species respectively. Out of the 15 endemic species, *Crotalaria rosenii* and *Polyscias farinosa* have been included in the IUCN red data list of Ethiopia and Eritrea qualifying for near threatened and vulnerable category respectively. In the Gerba Dima forest, at 625 m^2^ sample plot, species richness varied from 26 to 59 across the study plots. The Shannon diversity index also varied from 2.92 to 3.83 while evenness ranged from 0.89 to 0.95 in the study plots. The overall mean Shannon diversity index, species richness and evenness of the study area were 3.45, 41 and 0.93 respectively.

**Table 2:** Endemic species, their habit, IUCN status and geographical distributions

Species	Family	Habit	IUCN category	Altitude (m)
*Acanthopale ethio germanica*	Acanthaceae	Shrub	NE	2300_2600
*Aframomum corrorima*	Zingiberaceae	Herb	NE	1350_2000
*Arisaema mooneyanum*	Araceae	Herb	NE	2000_3450
*Bothriocline schimperi*	Asteraceae	Shrub	LC	1300_2820
*Clematis longicaudata*	Ranunculaceae	Liana	LC	1350_3300
*Crotalaria rosenii*	Fabaceae	Herb	NT	1350_2800
*Justicia bizuneshiae*	Acanthaceae	Herb	NE	1200_2100
*Millettia ferruginea*	Fabacae	Tree	LC	1000_2500
*Polyscias farinosa*	Araliaceae	Tree	VU	1600_2200
*Scadoxus nutans*	Amaryllidaceae	Herb	NE	1450_2300
*Solanecio gigas*	Asteraceae	Shrub	LC	1750_3350
*Tiliacora troupinii*	Menispermaceae	Liana	NE	1500_2100
*Urtica simensis*	Urticaceae	Herb	LC	1500_3400
*Vepris dainellii*	Rutaceae	Ttree	LC	1750_2500
*Vernonia rueppellii*	Asteraceae	Shrub	LC	2150_3000

Source: [24–32, 41] LC, Least Concern = A taxon is Least Concern when it has been evaluated against the criteria and does not qualify for Critically Endangered, Endangered, Vulnerable or Near Threatened; NE, Not evaluated = A taxon is Not Evaluated when it is has not yet been evaluated against the criteria; NT, Near Threatened=A taxon is Near Threatened when it has been evaluated against the criteria but does not qualify for Critically Endangered, Endangered or Vulnerable now, but is close to qualifying for or is likely to qualify for a threatened category in the near future; VU, Vulnerable = A taxon is Vulnerable when the best available evidence indicates that it meets any of the criteria, and it is therefore considered to be facing a high risk of extinction in the wild

### Community types and indicator species

**Fig. 4:**
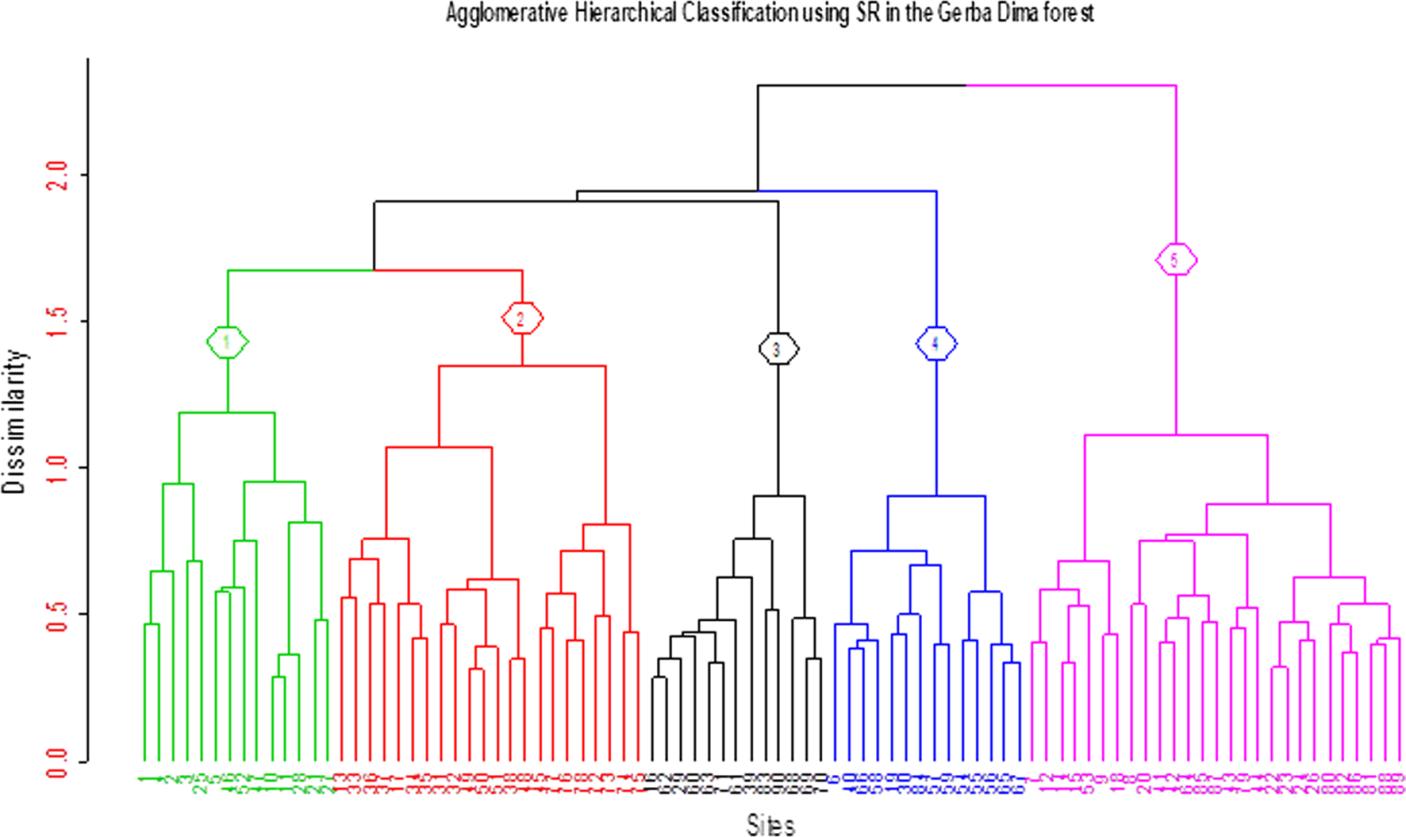
Dendrogram of the cluster analysis results of species abundance found in 90 plots

Five community types were derived from the hierarchical cluster analysis in combination with Multi-response Permutation Procedures (MRPP) and objective method of the whole data set (Fig. 4 and Table 3). From the output of MRPP, the test statistic T value for the five groups was −38.26 (P < 0.001) and the agreement statistic A was 0.13 while the output of objective method revealed a sharp bend at the fifth cluster.

**Table 3:** Synoptic cover value of plant in Gerba Dima Forest for species reaching ≥ 1% in at least one community

Cluster number	C1	C 2	C 3	C 4	C 5
Cluster size	14	22	13	14	27
*Allophyllus abyssinicus*	3.50	1.05	1.15	0.86	1.26
*Bersama abyssinica*	**3.71**	1.73	0.08	0.64	1.22
*Croton macrostachyus*	**7.50**	1.77	1.54	1.79	2.48
*Cordia africana*	2.79	0.55	0.00	0.00	0.63
*Olea welwitschii*	1.43	1.27	0.15	0.86	0.56
*Ehretia cymosa*	2.36	2.55	1.92	0.79	1.63
*Polyscias fulva*	1.64	1.36	1.85	0.93	1.33
*Apodytes dimidiate*	1.43	2.41	1.54	1.64	0.93
*Olea capensis*	3.14	**5.59**	1.54	1.93	1.04
*Syzygium guineense*	0.64	**5.91**	1.69	2.43	1.56
*Justicia schimperiana*	1.29	1.68	0.85	0.36	0.93
*Canthium oligocarpum*	0.29	1.05	0.62	0.50	0.52
*Cassipourea malosana*	0.79	1.55	1.08	0.43	0.56
*Combretum paniculatum*	0.86	1.14	0.54	0.43	1.04
*Dracaena steudneri*	1.29	3.09	0.92	1.50	1.22
*Elaeodendron buchananii*	0.64	1.00	0.38	0.00	0.37
*Oplismenus hirtellus*	2.43	4.50	3.00	2.64	2.85
*Rothmannia urcelliformis*	1.14	1.95	0.77	0.86	1.15
*Sapium ellipticum*	0.21	1.64	0.00	1.43	0.93
*Tectaria gemmifera*	0.93	1.36	1.23	1.29	0.81
*Brillantaisia madagascariensis*	1.43	2.73	3.00	2.79	2.48
*Dracaena afromontana*	1.00	3.05	**7.69**	0.86	0.78
*Ficus sur*	2.50	1.68	6.77	2.07	1.37
*Galiniera saxifrage*	1.00	1.05	2.31	1.43	1.56
*Hallea rubrostipulata*	1.07	0.00	1.31	0.00	0.00
*Macaranga capensis*	1.21	1.32	2.85	0.71	0.19
*Oxyanthus speciosus*	1.29	1.73	**7.01**	2.36	1.56
*Pouteria adolfi-friederici*	2.21	3.05	**7.31**	2.07	1.26
*Acanthopale ethio-germanica*	1.36	0.77	2.08	2.43	1.96
*Deinbollia kilimandscharica*	0.57	1.45	2.62	4.07	1.59
*Ilex mitis*	0.43	0.59	1.31	4.71	0.44
*Justicia bizuneshiae*	0.50	1.23	1.31	1.71	1.37
*Landolphia buchananii*	1.00	1.32	0.85	1.43	1.19
*Piper capense*	1.00	0.55	0.46	1.43	1.07
*Psychotria orophila*	0.93	1.36	0.85	1.36	0.85
*Pupalia micrantha*	0.64	1.36	0.23	1.86	0.93
*Schefflera abyssinica*	0.50	1.73	1.38	**7.29**	1.33
*Tiliacora troupinii*	1.00	1.23	1.08	1.29	1.07
*Vepris dainellii*	2.21	3.36	3.00	**8.43**	3.59
*Albizia gummifera*	3.07	2.50	2.69	2.07	**8.63**
*Clausena anisate*	1.79	1.86	1.31	1.43	2.11
*Hippocratea pallens*	0.64	1.91	1.23	1.50	1.93
*Lepidotrichilia volkensii*	0.57	2.59	1.23	2.43	3.30
*Maytenus gracilipes*	2.00	1.82	1.08	2.00	2.30
*Millettia ferruginea*	2.00	2.95	2.54	2.79	**7.89**

C1*, Croton macrostachyus—Bersama abyssinica*; C2*, Syzygium guineense—Olea capensis;* C3*, Dracaena afromontana—Pouteria adolfi-friederici;* C4, *Vepris dainellii Schefflera abyssinica* C5*, Albizia gummifera—Millettia ferruginea* community. Values in bold indicate the synoptic value of dominant species used in naming the plant communities

Community 1 (*Croton macrostachyus*—*Bersama abyssinica* community) was found in the altitudinal range of 1677–2020 m. a.s.l and slope from flat to 50%. Fourteen plots were associated with the community and has 2 indicator species with significant indicator values (P < 0.05) (Table 4).

**Table 4:** Indicator species of clusters in Gerba Dima forest with their significant P-value

Name of indicator species	Community type (C)	Indicator value	P-value
*Prunus Africana*	1	0.528	0.018*
*Rubus apetalus*	1	0.516	0.017*
*Flacourtia indica*	2	0.521	0.02*
*Pilea rivularis*	3	0.498	0.016*
*Elastostema monticolum*	3	0.467	0.039*
*Ritchiea albersii*	4	0.861	0.001***
*Trema orientalis*	4	0.677	0.001***
*Sapium ellipticum*	4	0.636	0.002**
*Vernonia hochstetteri*	4	0.538	0.014*
*Zehneria scabra*	5	0.581	0.002**
*Zehneria minutiflora*	5	0.552	0.005**
*Urera hypselodendron*	5	0.478	0.017*
*Vernonia wollastonii*	5	0.423	0.045*

C1*, Croton macrostachyus*-*Bersama abyssinica*; C2*, Syzygium guineense-Olea capensis;* C3*, Dracaena afromontana-Pouteria adolfi-friederici;* C4, *Vepris dainellii Schefflera abyssinica* C5*, Albizia gummifera-Millettia ferruginea* community. * = (p < 0.5), ** =(p < 0.01), *** = (p < 0.001)

Community 2 (*Syzygium guineense—Olea capensis* community) was distributed from 1699 to 2240 m a.s.l. and slope ranging from flat to 60%. It comprises of 22 plots and twenty species were associated with this community as indicator species where one of the indicator species exhibit significant indicator values (P < 0.05) (Table 4).

Community 3 (*Dracaena afromontana- Pouteria adolfi-friederici* community) was found in the altitudinal range of 1761–2000 m. a.s.l and slope from flat to 25%. Thirteen plots were associated with the community community and seven species were associated with this community as indicator species while two of the indicator species showed significant indicator values (P < 0.05) (Table 4).

Community 4 (*Vepris dainellii—Schefflera abyssinica* community) was distributed in the altitude range of 1720–2060 m a.s.l. and the slope gradient varies flat to 60%. It comprised of 14 plots, eight species were associated with this community as indicator species, while four of the indicator species exhibited significant indicator values (P < 0.05) (Table 4).

Community 5 (*Albizia gummifera—Millettia ferruginea* community) was found in the altitudinal range of 1728–2014 m. a.s.l and slope from flat to 50%. Twenty-seven plots were associated to the community. Eight species are associated with this community as indicator species and four of the indicator species exhibited significant indicator values (P < 0.05) (Table 4).

From computation of vegetation data in the study area Shannon-Weiner diversity and evenness, indices for the five community types showed the output in Table 5.

**Table 5:** Species richness, evenness and diversity indices of plant community types

Community	Species richness	Shannon diversity index (H')	Shannon Evenness
1	138	4.40	0.89
2	144	4.27	0.86
3	107	3.99	0.85
4	104	4.05	0.87
5	140	4.19	0.85

### Relationship between community types and environmental factors

Heterogeneity or homogeneity of vegetation data test using DCA resulted in short length (gradient) of DCA first axis i.e., < 3 (2.22) which indicate the presence of lower species turnover or homogeneous vegetation data due to the linear relationship between species and environmental variables. The result of Monte Carlo test showed that out of 14 environmental variables, seven were found to be significant in explaining patterns of plant community distribution. From the seven significant environmental factors, the vif values of sand and silt were higher than 5. Sand and Silt are highly correlated with at least one of the other variables in the model. One solution in dealing with collinearity is to remove some of the violating variables from the model and thus the one with higher vif value (sand) was eliminated. The result of RDA ordination showed that comparatively, the gradient of altitude and potassium was highly correlated on axis one and gradient of disturbance in axis two. The other factors were correlated with the five axes with a different value of correlation. The eigenvalue for axis one, two and three were 10.65, 8.06, and 6.32 respectively. Cumulative proportion variance explained by the first five RDA axis of the joint biplot was 93.9%. The proportion of variation explained by five RDA axis also shows a decline towards the successive higher axis (Table 6).

**Table 6:** Biplot score for constraining variables and their correlation with the RDA axis, eigenvalues and proportion of variance explained

Environmental variables	RDA1	RDA2	RDA3	RDA4	RDA5
Disturbance	0.089	−0.68	−0.157	0.522	0.453
Altitude	0.880	0.42	−0.054	−0.018	0.218
SILT	0.084	−0.40	−0.361	−0.323	−0.370
EC	−0.053	0.31	−0.867	0.094	−0.023
OM	−0.094	0.27	−0.208	−0.357	0.844
K	0.703	−0.27	−0.003	−0.381	−0.246
Eigenvalue	10.6445	8.0649	6.3168	5.0057	3.02318
Proportion explained	0.3024	0.2291	0.1794	0.1422	0.08588
Cumulative proportion	0.3024	0.5315	0.7109	0.8531	0.93902

**Fig. 5:**
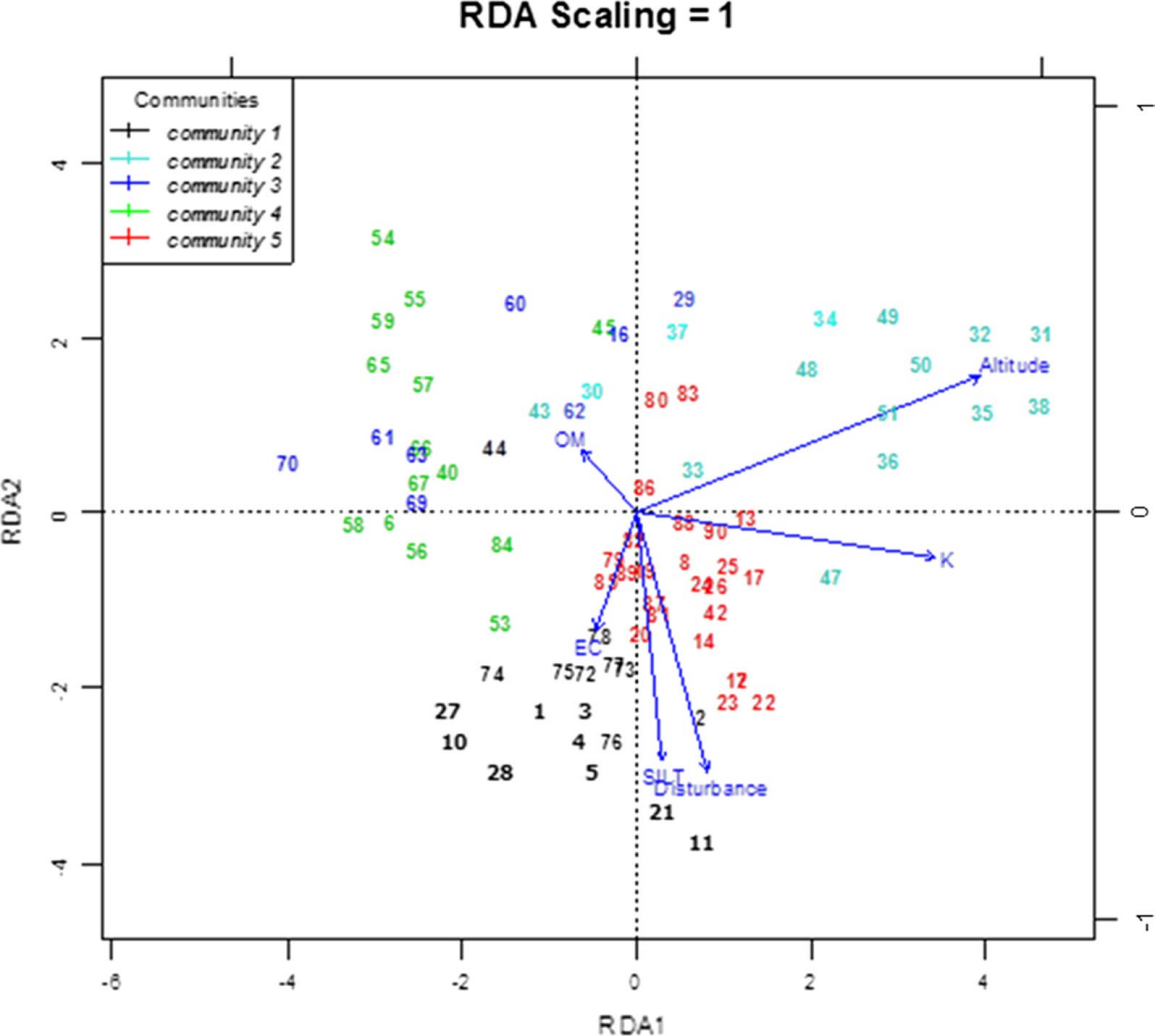
RDA ordination biplot of 90 quadrats and 6 environmental variables of plant communities

RDA ordination of the study plots of Gerba Dima forest formed five groups or community based on the species composition. These five community types were segregated following the arrows of the environmental variables. Community 3 and community 4 are found in mid altitude area. Community two mostly occur at the higher altitude while species in community 1 and community 5 are distributed at the lower altitude and higher EC. Silt, Disturbance and potassium axes were strongly influencing the distribution of community five. Organic matter arrow has strongly influenced the distribution of species in community three and four (Fig. 5). The ANOVA test indicated that the five community types differ significantly from each other with regard to EC and K. The result of Tukey’s pair-wise comparison test indicates that community 4 and 1 differ significantly with respect to Disturbance and K while community 2 and 3 showed significant differences with respect to EC.

## Discussion

### Floristic composition and diversity of Gerba Dima forest

The existence of diversified flora of Gerba Dima forest was in line with the general pattern of high species diversity in the tropical montane forests. According to Gentry [42], tropical forests are among ecosystems that harbour high species diversity of the globe. East African montane forests of Ethiopia, Kenya, Tanzania and Uganda are among the most diverse and richest African regions with regard to flora composition and endemic plant taxa [43–45]. Asteraceae, Acanthaceae, Rubiaceae, Fabaceae and Euphorbiaceae are the five dominant families, which contribute more than 27% of the total species in the study forest. These dominant families were also reported as top ten species rich families in many Neotropical forests and Asia [42]. Except for Rubiaceae, these families are also among the top ten species rich families in the flora area [46]. The dominance of the above families together with Rubiaceae was also reported in other moist afromontane forests of southwestern Ethiopia [47–49]. Thus, the dominance of these families in the Gerba Dima forest agreed to their general dominance in the flora area and tropical forests. The dominance of these families in the study area could be attributed to their successful colonization to the landscape owing to their efficient pollination, dispersal and germination mechanisms [50]. For instance, many species of Asteraceae have umbrella shape structures adapted for air dispersal and increase their opportunity for their successful establishment [50].

Among the growth forms, herbs constitute more than 42% of recorded species. The prevalence of herbs could be attributed to the presence of canopy gap because of anthropogenic disturbance. Disturbance of forest in the form of selective cutting of trees favours the growth of herbaceous species in the forest understory. Under normal circumstances, the forest floor (herbaceous layer) of Afromontane rainforests is usually dark and poor in species composition owing to the closed canopy of the forest that prevents light from reaching the ground [51].

The higher value of Shannon diversity index and evenness indicates that the study forest has high species diversity with more even distribution of the species within the study plots. Species diversity increases when the populations have more even abundances and vice versa [40]. High Shannon evenness in the Gerba Dima forest indicates little dominance by any single species but the repeated coexistence of species over all the plots or sites. Therefore, the implication of evenness values is that, when there is a high evenness value in a given forest, the location of conservation sites might not be of much importance compared to when the evenness value of the forest is low.

To give a general impression of the species richness of Gerba Dima Forest, the results of the present study were compared with results from other Moist Afromontane forests in Ethiopia. The species richness of Gerba Dima forest is higher than some moist afromontane forest of Ethiopia such as Masha forest (130 species) [48], Belete forest (157 species) [52], Gelesha forest (157 species) [53], Agama forest (162 species) [49] and more or less similar in species richness with some other moist afromontane forest of Ethiopia such as Komto forest (180 species) [54] and Jibat forest (183 species) [55]. However, the species richness of Gerba Dima forest was much lower than the values reported for few other moist afromontane forest of Ethiopia which include Bonga forest (243 species) [47], Yayu forest (220 species) [56] Mana Angetu forest (212 species) [57] (Magada forest (197 species) [58] and Gesha and Sayilem forest (300 species [59].

The difference in species richness among the compared forests could be attributed to the variations of forest sites with regard to geographical location, altitude, anthropogenic impact, rainfall and other climatic, physiographic and edaphic factors [60, 61]. Climatic and physiographic factors have a wide range of effect on the diversity of plant species across the land escape whereas suitable environmental conditions and biotic factors influence diversity at the site level [62, 63]. Species composition of forests is also influenced by regeneration success and competition among species [64].

### Plant community types in Gerba Dima forest

The output of Multi-response Permutation Procedures (MRPP) results in T statistics having more negative value with significant P-value (T = − 38.26, P < 0.001) and an agreement statistic A (0.13) confirming the distinctness of clusters. The test statistic T describes the separation between the groups. The more negative T value, the stronger the separation. From the result of this study, the null hypothesis of no difference among groups can be rejected. The five groups occupy different regions of species space, as shown by the strong chance correction within the group (A) and test statistic (T) and thus confirm the existence of 5 distinct plant communities in the Gerba Dima forest [37]. The five plant communities showed a slight variation in their species richness, diversity and evenness. Relatively community types 1, 2 and 5 were the richest with respect to species richness and diversity while community types 3 and 4 the lowest. The differences in species richness among the five communities could mainly be attributed to the dissimilarities of the communities in terms of location, altitude, human impact, rainfall, and other biotic and abiotic factors. According Eilu and Obua to [65], different altitudes and slopes influence species richness and dispersion behaviour of tree species. Altitude and climatic variables like temperature and rainfall are also other determinant factors that affect species richness [66].

### Plant community—environmental variables relationship

In the current study, the multivariate analyses (both Ordination and cluster analysis) were consistent in showing the patterns of floristic grouping within the studied forest and hence the two methods are complementary. The variable with the highest score (0.88) associated with axis one was the altitude. Therefore, altitude was the most important variable in weighting axis one and to interpret or explain the axis. Similar studies conducted in other Afromontane forests of Ethiopia also confirm the importance of altitude as a major determinant of vegetation distribution along altitudinal gradients [57, 67, 68]. Altitudinal change leads to changes in humidity, temperature, soil type, and other factors that influence the growth and development of plants which in turn determine the patterns of vegetation distribution [69, 70].

Potassium followed by altitude was also the most important constraining variable in weighing axis one in the ordination. In the sandy soil, plant-soil feedback effects were most strongly correlated with potassium. Although most studies investigating abiotic plant-soil interactions have focused on nitrogen and phosphorus dynamics, in sandy soils with little clay content, potassium could be a limiting factor for plant growth [71, 72]. In particular, a growth of forbs can be highly dependent on potassium [71] and hence potassium at least affects the distribution of these species. In the same way, the disturbance was the most important variable in weighting axis two. Disturbance affects the distribution of plant communities by hampering natural regeneration and seedling establishment in tropical forests [73]. Disturbance also favours the growth of herbaceous plant species by improving the availability of light conditions in the ground layer as it widens the canopy gap [74] and thus affects the distribution of communities with these species. An analysis of variance (ANOVA) performed to see any significant variation among the community types of Gerba Dima forest with respect to non-collinear significant environmental variables indicated that the five community types differ significantly from each other with regard to EC and K. Similarly, result of Tukey’s pair-wise comparison test indicates that community 4 and 1 differ significantly with respect to Disturbance and K while community 2 and 3 showed significant difference with respect to EC.

## Conclusions

Description of the floristic diversity of species in the Gerba Dima forest revealed the presence of high species diversity and richness. Of the species recorded in this forest, 15 (8.3%) species were endemic to Ethiopia. However, the percentage of endemic species in the study forest is lower than the proportions generally expected in the Afromontane forest of Ethiopia and this is attributed to the low endemicity feature of forests in South-western Ethiopia. In this study, five community types were identified and altitude was the major environmental variable in determining the community types. The existence of high species diversity and a number of endemic plant species in the study forest shows the potential of the area for biodiversity conservation. Thus, all Stakeholders including Oromia Forest and wildlife enterprise (OFWE) and the regional government should work to designate the forest as a biosphere reserve and being registered under UNESCO.

## Supplementary Information

**Additional file 1:** Dominant families with their respective species number of Gerba Dima Forest.

## Data Availability

We have also included part of the data used in this research and attached as Additional files 1.

## Abbreviations

DCA: Detrended Correspondence Analysis
RDA: Redundancy Analysis
Vif: Variance inflation factor
MRPP: Multi-response Permutation Procedures
