# Supplementary material for: Plant community analysis along environmental gradients in moist afromontane forest of Gerba Dima, South-western Ethiopia
Source: BMC Ecol Evol. 2022 Feb 7;22:12. doi: 10.1186/s12862-022-01964-4 (PMC8819878; doi:10.1186/s12862-022-01964-4)
Supplement: Supplementary file 1 — Additional file 1: Dominant families with their respective species number of Gerba Dima Forest. [file 12862_2022_1964_MOESM1_ESM.docx]

**Additional file 1. Dominant families with their respective species number of Gerba Dima Forest**

| **Familiies** | **Number of**  **Genera** | **Number of**  **species** | **% Richness** | **Familiies** | **Number of**  **genera** | **Number of**  **species** | **% Richness** |
| --- | --- | --- | --- | --- | --- | --- | --- |
| Acanthaceae | 9 | 11 | 6.11 | Marattiaceae | 1 | 1 | 0.56 |
| Adiantaceae | 1 | 1 | 0.56 | Melianthaceae | 1 | 1 | 0.56 |
| Alengeaceae | 1 | 1 | 0.56 | Melistostomaceae | 1 | 1 | 0.56 |
| Amaranthaceae | 3 | 3 | 1.67 | Menispermaceae | 2 | 2 | 1.11 |
| Amaryllidaceae | 1 | 1 | 0.56 | Moraceae | 2 | 5 | 2.78 |
| Apocynaceae | 1 | 1 | 0.56 | Musaceae | 1 | 1 | 0.56 |
| Aquifoliaceae | 1 | 1 | 0.56 | Myrsinaceae | 2 | 2 | 1.11 |
| Araceae | 3 | 3 | 1.67 | Myrtaceae | 1 | 1 | 0.56 |
| Araliaceae | 2 | 4 | 2.22 | Oleaceae | 3 | 4 | 2.22 |
| Asclepidiaceae | 1 | 2 | 1.11 | Orchidaceae | 2 | 2 | 1.11 |
| Asparagaceae | 1 | 3 | 1.67 | Piperaceae | 2 | 3 | 1.67 |
| Aspleniaceae | 1 | 6 | 3.33 | Pittosporaceae | 1 | 1 | 0.56 |
| Asteracea | 6 | 11 | 6.11 | Poaceae | 2 | 2 | 1.11 |
| Boraginaceae | 2 | 2 | 1.11 | Polypodiaceae | 2 | 2 | 1.11 |
| Capparidaceae | 1 | 1 | 0.56 | Pteridaceae | 1 | 2 | 1.11 |
| Caryophyllaceae | 1 | 1 | 0.56 | Ranuaculaceae | 3 | 3 | 1.67 |
| Celastraceae | 3 | 5 | 2.78 | Rhaminaceae | 1 | 2 | 1.11 |
| Combreataceae | 1 | 1 | 0.56 | Rhizophoraceae | 1 | 1 | 0.56 |
| Commelinaceae | 1 | 1 | 0.56 | Roseaceae | 3 | 4 | 2.22 |
| Convolvulaceae | 1 | 1 | 0.56 | Rubiaceae | 11 | 11 | 6.11 |
| Crassulaceae | 1 | 1 | 0.56 | Rutaceae | 3 | 3 | 1.67 |
| Cucurbitaceae | 3 | 4 | 2.22 | Sapindaceae | 2 | 2 | 1.11 |
| Cyatheaceae | 1 | 1 | 0.56 | Sapotaceae | 1 | 1 | 0.56 |
| Cyperaceae | 2 | 3 | 1.67 | Simaroubaceae | 1 | 1 | 0.56 |
| Dracenaceae | 1 | 3 | 1.67 | Solanaceae | 1 | 2 | 1.11 |
| Dryopteridaceae | 3 | 3 | 1.67 | Sterculiaceae | 1 | 1 | 0.56 |
| Euphorbiaceae | 6 | 7 | 3.89 | Tectariaceae | 2 | 2 | 1.11 |
| Fabaceae | 8 | 9 | 5.00 | Tilaceae | 1 | 1 | 0.56 |
| Flacourtiaceae | 1 | 1 | 0.56 | Ulmaceae | 2 | 2 | 1.11 |
| Hemionitidaceae | 1 | 1 | 0.56 | Urticaceae | 4 | 4 | 2.22 |
| Icaccinaceae | 1 | 1 | 0.56 | Verbenaceae | 1 | 1 | 0.56 |
| Lamiaceae | 4 | 4 | 2.22 | Vitaceae | 1 | 1 | 0.56 |
| Lobeliaceae | 1 | 1 | 0.56 | Vittariaceae | 1 | 1 | 0.56 |
| Malvaceae | 2 | 2 | 1.11 | Zingiberaceae | 1 | 2 | 1.11 |
| Meliaceae | 4 | 4 | 2.22 |  |  |  |  |
